# Type D Syndrome of Inappropriate Antidiuretic Hormone Secretion in a Schizophrenia Patient with Polydipsia

**DOI:** 10.4137/jcnsd.s2330

**Published:** 2009-04-20

**Authors:** Takahira Yamauchi, Manabu Makinodan, Tomohisa Nagashima, Kuniaki Kiuchi, Yoshinobu Noriyama, Toshifumi Kishimoto

**Affiliations:** Department of Psychiatry, Nara Medical University Faculty of Medicine, Kashihara, Japan.

**Keywords:** polydipsia, water intoxication, schizophrenia, hyponatremia, plasma osmolality

## Abstract

A 55-year-old man with schizophrenia developed water intoxication due to primary polydipsia. His manner of antidiuretic hormone secretion was investigated by water loading and infusion of hypertonic saline to clarify the form of the syndrome of inappropriate antidiuretic hormone secretion. The plasma antidiuretic hormone level, which may be involved in the occurrence of water intoxication, was consistently low in this patient, and linked to type D syndrome of inappropriate antidiuretic hormone secretion, designated “hypovasopressinemic antidiuresis”. Although this type is not common, it should be considered as a pathophysiology for water intoxication in schizophrenia patients.

## Introduction

The syndrome of water intoxication is a clinical entity that occurs in patients with chronic mental disorders. The essential mechanism of this syndrome is rapidly progressive profound hyponatremia due to polydipsia with excessive water intake. Since excessive water intake alone is rarely sufficient to produce hyponatremia, water intoxication has been linked to the syndrome of inappropriate secretion of antidiuretic hormone (SIADH).^1^,^2^ We present here a case of water intoxication in a schizophrenia patient with polydipsia who was also studied during a period of stability with provocative physiological challenges. The results of this testing indicate that this is type D SIADH,^3^ and that this abnormality was still present during a period of electrolyte stability.

## Presentation of Case

The patient was a 55-year-old male diagnosed with schizophrenia, residual type based on DSM-IV criteria. The onset of psychotic symptoms occurred at 32 years of age, and therapy with antipsychotics was initiated. The patient had suffered from several psychotic relapses and been hospitalized four times. His water intake per day had been about 8–10 L from 40 years of age, and he became aware of dizziness and calf cramps from 45 years of age. When the patient was 55 years of age, he suffered from leg weakness and was admitted to Nara Medical University Hospital. On admission, he had severe hyponatremia in his plasma (plasma sodium, 116 mEq/l) and had to be treated by continuous hemodiafiltration for acute renal failure.

About two months later, when his hyponatremia and renal failure had completely improved, we carried out water loading and infusion of hypertonic saline^4^ to differentiate his type of SIADH.

## Procedure

### Water loading

To decrease the osmolality, oral water loading (20 ml per kilogram of body weight) was administered over a period of 15 minutes, and blood samples were obtained before loading, and at 30, 60, 90 and 120 minutes after loading.

### Infusion of hypertonic saline

To increase the osmolality, 5% hypertonic saline was infused intravenously at a regular rate (0.1 ml per kilogram per minute) for 120 minutes using a constant-infusion pump, and blood samples were obtained before loading, and at 30, 60, 90 and 120 minutes after the beginning of loading.

The plasma osmolality (Posm) and plasma antidiuretic hormone (ADH) levels were measured in the core laboratory of Nara Medical University Hospital. Briefly, Posm was determined in fresh plasma by freezing point depression, while the plasma ADH level was measured in duplicate by a double antibody radioimmunoassay technique.

## Results

**Figure 1:**
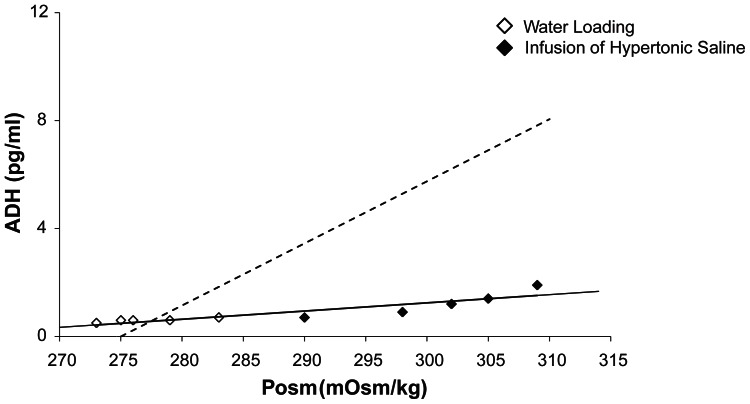
Relationship between ADH and Posm. Open rhombuses: water loading; solid rhombuses: infusion of hypertonic saline. The regression line in the patient is Y = 0.0303X – 7.8434. The dotted regression line in population samples is Y = 0.23X – 63.25, the data of which were obtained from healthy controls and patients without electrolyte abnormalities (n = 85, in total).^15^

Table 1 shows the Posm values and plasma ADH levels for water loading and infusion of hypertonic saline. The Posm values were relatively low (273–283 mOsm/kg) and the plasma ADH levels were also low (0.4–0.6 pg/ml) for water loading. The plasma ADH levels linearly increased relative to Posm in the infusion of hypertonic saline. However, the rate of increase in ADH to Posm was lower than that in population samples (Fig. 1).

## Discussion

Primary polydipsia and water intoxication are uncommon complications of chronic psychiatric patients. The pathophysiologies of these disorders are hyponatremia and brain edema followed by nausea, dizziness, ataxia, seizure and coma. Barahal^5^ documented the first case of water intoxication in a schizophrenia patient. In other studies, the ratio of primary polydipsia in long-term inpatients with schizophrenia was found to be >20%^6^ while that of hyponatremia in psychiatric inpatients was 10.5%.^7^

Primary polydipsia generally develops in the following three phases:^8^ 1, polydipsia and polyuria; 2, hyponatremia; and 3, water intoxication. Although the pathophysiologies of polydipsia and hyponatremia remain unclear, they are thought to be induced by SIADH. SIADH is a state of ADH hypersecretion relative to Posm, followed by inappropriate free-water retention and decreased plasma sodium. According to Zerbe et al.^3^ the osmoregulation abnormality in SIADH takes several different forms. In some cases, ADH is secreted in a random fashion independently of the Posm (type A). A second form is “reset osmoset” (type B). In this type, ADH is completely responsive to osmotic influences. However, because the threshold or set of the system is subnormal, ADH osmoregulation remains preserved but the initiation of relapse is premature. A third form is characterized by ADH that appears to be fixed at inappropriately high levels under hypotonic conditions, but increases normally when the Posm exceeds the usual threshold values (type C). The last type is termed “hypovasopressinemic antidiuresis” (type D). Type D patients exhibit no detectable abnormalities in ADH secretion, yet they can neither maximally dilute their urine nor excrete a water load normally. This type of abnormality in the water balance could be due to either an increase in renal sensitivity to ADH or the presence of another antidiuretic substance, the details of which are not yet understood.

According to the dynamic state of Posm and ADH, the SIADH in the present patient was classified as type D. This conclusion was based on several findings. First, the ADH levels were not random relative to Posm for water loading and infusion of hypertonic saline (Table 1, Fig. 1), indicating that the SIADH was not type A. Second, the ADH levels was lower than that in population samples for infusion of hypertonic saline (Table 1, Fig. 1), indicating that the SIADH was not type B. Third, the ADH levels were low to the same level of those of population samples for water loading (Table 1, Fig. 1), indicating that the SIADH was not type C. Therefore, the SIADH in the present patient was type D, which is characterized by the deficit of renal diluting function even in normal plasma ADH levels. This is because the ADH levels were consistently low, but the relationship between the Posm and the ADH was linear, and the urine was not diluted on admission even during water intoxication (urine osmolality, 291 mOsm/kg). As for the consistently low plasma ADH levels, Goldman et al. reported that polydipsia could make ADH response slightly blunt in schizophrenia patients.^9^ These data suggest that the hypo-osmolar state of his plasma could have been produced by activated renal function with ADH despite the consistently low level of ADH (hypovasopressinemia), as we previously reported.^10^,^11^

Although a number of reports have investigated the different forms of SIADH in schizophrenia patients,^12^–^14^ only one previous report identified type D SIADH in a schizophrenia patient with polydipsia by both water loading and infusion of hypertonic saline.^11^ The present report sheds further light on type D SIADH in polydipsic schizophrenic patients and represents a reminder of its occurrence.

## Figures and Tables

**Table 1:** Data for water loading and infusion of hypertonic saline (Osmolalities and ADH levels in plasma).

	Time (min)	0	30	60	90	120
Water loading	Posm (mOsm/kg)	283	279	273	275	276
	ADH (pg/ml)	0.7	0.6	0.5	0.6	0.6
Infusion of hypertonic saline	Posm (mOsm/kg)	290	298	302	305	309
	ADH (pg/ml)	0.7	0.9	1.2	1.4	1.9
